# Hormonal Biomarkers for Evaluating the Impact of Fetal Growth Restriction on the Development of Chronic Adult Disease

**DOI:** 10.1055/s-0039-1683904

**Published:** 2019-04-02

**Authors:** Elizabeth Soares da Silva Magalhães, Maria Dalva Barbosa Baker Méio, Maria Elisabeth Lopes Moreira

**Affiliations:** 1 Clinical Research Unit, Fundação Oswaldo Cruz, Rio de Janeiro, RJ, Brazil

**Keywords:** fetal growth restriction, developmental origins of health and disease, growth, development, biomarkers, restrição de crescimento fetal, origens desenvolvimentistas da saúde e doença, crescimento, desenvolvimento, biomarcadores

## Abstract

The hypothesis of fetal origins to adult diseases proposes that metabolic chronic disorders, including cardiovascular diseases, diabetes, and hypertension originate in the developmental plasticity due to intrauterine insults. These processes involve an adaptative response by the fetus to changes in the environmental signals, which can promote the reset of hormones and of the metabolism to establish a “thrifty phenotype”. Metabolic alterations during intrauterine growth restriction can modify the fetal programming. The present nonsystematic review intended to summarize historical and current references that indicated that developmental origins of health and disease (DOHaD) occur as a consequence of altered maternal and fetal metabolic pathways. The purpose is to highlight the potential implications of growth factors and adipokines in “developmental programming”, which could interfere in the development by controlling fetal growth patterns. These changes affect the structure and the functional capacity of various organs, including the brain, the kidneys, and the pancreas. These investigations may improve the approach to optimizing antenatal as well as perinatal care aimed to protect newborns against long-term chronic diseases.

## Resumo

A hipótese das origens fetais de doenças em adultos propõe que distúrbios crônicos metabólicos, incluindo doenças cardiovasculares, diabetes e hipertensão, se originam na plasticidade do desenvolvimento devido a insultos intrauterinos. Estes processos envolvem uma resposta adaptativa do feto a mudanças nos sinais ambientais que podem promover a redefinição dos hormônios e do metabolismo para estabelecer um “fenótipo poupador”. Alterações metabólicas durante a restrição de crescimento intrauterino podem modificar a programação fetal. A presente revisão não-sistemática pretendeu resumir referências históricas e atuais que indicassem que as origens desenvolvimentistas da saúde e doença (DOHaD, na sigla em inglês) ocorrem como consequência de alterações nas vias metabólicas materna e fetal. O propósito é destacar as potenciais implicações de fatores de crescimento e adipocinas na “programação do desenvolvimento”, que poderia interferir no desenvolvimento, controlando os padrões de crescimento fetal. Estas alterações afetam a estrutura e a capacidade funcional de inúmeros órgãos, incluindo o cérebro, os rins e o pâncreas. Estas investigações podem melhorar a abordagem para otimizar os cuidados pré-natais e perinatais, com o objetivo de proteger os recém-nascidos contra doenças crônicas em longo prazo.

## Introduction

Intrauterine growth restriction is associated with increased morbidity and mortality in the perinatal period, with implications for later adult life diseases.^1^ The pioneering studies by Barker have drawn attention to the association between growth in intrauterine life and the development of chronic diseases, such as arterial hypertension and cardiovascular diseases.^2^ Since then, several studies have demonstrated adaptive mechanisms that occur during growth restriction. These chronic diseases may occur in combination or not, and have imbalance of homeostasis involving the common regulatory hormonal axis, with potential effects on fetal development as well as on long-term adult health.^3^ ^4^ ^5^

Intrauterine insults can modify the trajectory of fetal growth, resulting in low birthweight.^5^ ^6^ These processes involve an adaptative response by the fetus to changes in the environmental signals that can promote the reset of hormones and of the metabolism to establish what is known as thrifty phenotype. These adaptative mechanisms alter the structure and the function of various organs, and favor the development of insulin resistance. Although the thrifty phenotype favors survival, it may have a developmental impact by affecting the hormonal regulatory axis that regulates fetal growth.^7^ These processes involve hormonal factors, such as insulin-like growth factor (IGF) system and insulin, which participate in the regulation of fetal growth, and which can modify the fetal development during intrauterine growth restriction.^3^ ^4^ ^5^

These adaptations prepare the fetus for extrauterine life. This phenomenon, known as “programming”, refers to the fact that stimuli may generate permanent changes that persist throughout life. Programming is not just limited to the intrauterine environment, but extends into childhood and adolescence, when systems continue to adapt to various pathological challenges, therefore originating the concept of fetal development programming.^8^ ^9^ ^10^

## Fetal Origins of Adult Disease: A Historical Perspective

The fetal origins of cardiovascular and metabolic chronic diseases in childhood and adulthood was originally proposed by Barker et al,^2^ who demonstrated that the intrauterine conditions of nutritional restriction could have a permanent effect on the metabolism and on the physiology of the developing organs.^8^ These studies stimulated researchers to investigate the fetal origins of adult diseases and the formation of an international society, the International Society for Developmental Origins of Health and Disease (DOHaD) (https://dohadsoc.org/), which focus its studies in the first stages of the human development, looking for solutions for the prevention of chronic diseases.

For most organs and systems, the critical period of plasticity is during the intrauterine development. There are specific developmental periods in which the fetus, due to plasticity, alters the structure and the function of organs to promote survival. This phenomenon, known as “programming”, prepares the fetus for extrauterine life and is associated with persistent effects in the structure and in the function of organs such as the kidney and the liver. Beyond that, the concepts of critical windows and of plasticity show how the period of the first 1,000 days of life can determine the health for adult life. Epidemiological, experimental, and clinical studies support this theory.^11^ ^12^

The first epidemiological findings on the hypothesis of DOHaD arose from historical cohorts. In these studies, low birthweight was associated with increased rates of chronic metabolic diseases.^11^ Barker et al^2^ proposed this relationship by evaluating mortality from coronary heart disease in Hertfordshire. The Hertfordshire and Helsinki cohorts from the 1930s and 1940s linked poor fetal growth with coronary heart disease, hypertension, and insulin resistance in adult men and women. Specifically, a lower weight at the age of 1 year old was associated with an increased death rate from coronary heart disease.^2^ ^13^

The exposure to intrauterine starvation during the first 2 trimesters of gestation led to a higher prevalence of cardiovascular diseases and of type 2 diabetes mellitus (DM) in adulthood, as demonstrated by the Dutch Famine Study. During the period known as “Dutch Famine” of 1944 and 1945, at the end of World War II, the energy consumption of pregnant women was reduced to around between 500 and 1,000 calories. The German government began to reduce the caloric supply for residents in the west of the Netherlands. The daily nutritional intake, which was of ∼ 1,800 calories in December 1943, fell abruptly to < 1,000 calories in late November 1944, and varied between 500 and 800 calories in the period from December 1944 to May 1945, when the liberation of the Netherlands took place.^14^ ^15^ ^16^

These findings from historical cohorts suggest that one of the mechanisms linking poor weight gain in infancy with coronary heart disease is altered liver function, and also hormonal and lipid profile alterations.^17^ ^18^ Low birthweight associated with insulin resistance are risk factors for both coronary diseases and its 2 major risk factors: type 2 DM and hypertension.^19^ ^20^ The consequences of metabolic disorders may be more severe in newborns with lower birthweight due to growth restriction.^21^ In the studies that explore the mechanisms underlying these associations, it was found that low birthweight was associated with reduced high-density lipoprotein (HDL) cholesterol levels, increased low-density lipoprotein (LDL) cholesterol and triglyceride levels, and was related to glucose intolerance, as well as to insulin resistance.^15^ ^22^ In addition to pathophysiological changes, it was found that people who develop chronic diseases have a different pattern of growth.

Moreover, the increased prevalence of cardiovascular diseases and of DM 23 24 cannot be explained solely by genetics, and, in part, it is well established that environmental factors are determinant. These periods of social and economic transformations, including periods of war, industrialization processes, and changes in dietary habits, have favored an increase in the prevalence of and an epidemic of nontransmissible chronic diseases in the world. These environmental factors affect the programming of fetal development.^25^ ^26^

## Endocrine Control of Growth and Fetal Development

Intrauterine growth is regulated by genetic potential and is controlled and modified by hormonal, nutritional, and immunological factors.^27^ These processes during development are dependent on the adequate supply of oxygen and substrates, glucose, and amino acids through the maternal circulation via the placenta. It also involves endocrine signals including the human placental lactogen and placental growth hormones, which induce resistance to maternal insulin and facilitate the mobilization of nutrients for fetal growth. These hormones integrate the metabolic adaptations of gestation for the control of maternal metabolism and fetal growth.^28^ ^29^

Hormones that stimulate growth also regulate the fetal development. These hormones are released in response to fetal insulin, which is determined by the transfer of glucose from the placenta.^30^ Glucose transport to the fetus across the placenta takes place via glucose transporters in the opposing faces of the barrier layer, the microvillous, and the basal membranes of the syncytiotrophoblast. These transporters are inversely regulated by glucose concentration, and positively regulated by IGF-1 and by placental growth hormone.^31^ Fetal insulin and IGFs are thought to have a central role in the regulation of growth.^32^ ^33^

The development of the fetal endocrine system can be adapted to a range of challenges, including hypoglycemia during growth restriction due to malnutrition, or inflammatory insults.^34^ As a consequence, the subsequent growth trajectory and endocrine organ function of the fetus can be permanently reset. These developmental adaptations may not result in clinically apparent sequelae. However, these processes of adaptation are strongly associated with a range of adult metabolic diseases, including diabetes, obesity, and coronary heart diseases.^27^ ^35^

## Fetal Growth Restriction and Hormonal Biomarkers

Metabolic alterations involving placental hormones, IGF-1, and leptin during gestation occur due to growth restriction, which compromise the fetal metabolism.^36^ These hormones are involved in the regulation of energy balance and may have a role in the regulation of growth and development in the neonatal period and infancy, as well as long-term effects on the metabolic programming. Intrauterine growth restriction affects concentrations of growth factors and hormones.^32^ ^37^ Low levels of IGF-1 and/or of insulin-like growth factor-binding protein 3 (IGFBP-3) in the fetal blood have been described.^38^ Therefore, the alterations of these factors and maternal arterial resistance may contribute to the impairment of placental circulation during growth restriction. These hormones may be associated with placental volume and blood flow indexes analyzed by Doppler studies.^39^ In these regulatory mechanisms, an imbalance of the insulin-IGF-1 axis and of leptin occurs, both in the maternal and fetal circulations.^40^ Higher levels of leptin were associated with low weight gain in the 1^st^ semester of postnatal life and with metabolic disorders.^41^ Maternal malnutrition and placental insufficiency have long-term effects on the expansion and function of fetal adipose tissue, now recognized as an endocrine organ.^42^ Levels of leptin correlate with fetal growth rates, and were characterized as an endocrine marker of fetal size.^43^ Fetal insulin and IGFs are thought to have a central role in the regulation of growth. Metabolic alterations during the fetal period can modify growth patterns, and are characterized as diagnostic markers in the identification of neonates with growth restriction.^40^ These regulatory loops of IGF-1, insulin, and leptin may affect the ontogeny and physiology of organs such as the kidneys, the lungs, and the pancreas.^44^ ^45^

## The Programming of Fetal Development: The thrifty phenotype

The adaptative mechanism to intrauterine nutritional restriction alters the metabolism and the physiology of developing organs.^46^ These events lead to a slowed growth rate, and the fetus saves glucose, avoiding the risks of hypoglycemia.^47^ Insulin resistance may be viewed as persistence of a fetal response by which blood glucose concentrations were maintained for the benefit of the brain and of the heart, but at the expense of glucose transport into the muscles and muscle growth.^48^ In these critical periods during the development, in which the fetus is more sensitive to environmental changes, its trajectory of growth is modified. These changes in development programming provide an adaptative advantage for this new adverse intrauterine environment, promoting a thrifty phenotype.^7^ ^49^

The hemodynamic response is the vasodilation in the vital organs to favor the supply of nutrients. The vasoconstriction occurs in peripheral organs, which may compromise the development of the pancreas, of the liver, and of the kidneys, responsible for the production of hormones that are essential for the maintenance of homeostasis.^48^ ^50^ Research in experimental models has demonstrated that IGF-1 and leptin are involved in the development and maturation of a number of organs, including the heart, the brain, the kidneys, and the pancreas.^44^ ^45^

Fetal growth restriction may impair the development of the kidney and reduce the nephron number, which is involved in the regulation of blood pressure. Insulin-like growth factors are important for the normal development of the kidneys.^51^ The development of nephrons extends into the 3^rd^ trimester of gestation, and renal growth below the ideal can have long-term consequences. In the pathophysiology of fetal growth restriction, the reduction of insulin and of IGF-1 levels causes a decrease in the proliferation of renal cells, which may affect the pressure of glomelluric capillaries. The compensatory hyperfiltration in the remaining nephrons results in glomerular and systemic hypertension, which can cause damage to the kidneys.^50^ The smaller size of the kidneys by the reduction of the number of nephrons has been described in fetuses with intrauterine growth restriction.^52^ Therefore, the actions of these hormones can alter the programming of the nephrogenesis during fetal growth restriction.

In the development of the pancreas, perinatal insults can affect processes of proliferation and of differentiation in the first months of gestation. In placental insufficiency, there is a reduction in IGF-1 levels that compromise the function of pancreatic beta cells. This was evidenced by decreased insulin, glucose, insulin/glucose ratios, and β-cell number and function.^53^ ^54^ Leptin also inhibits the secretion of insulin and participates in the regulation of the metabolism of glucose.^53^ Alterations in maternal-placental-fetal leptin exchange may modify the development of the fetus due to deregulated energy stores; they can also affect the development of the pancreas, contributing to an increased risk of developing diseases in adulthood.^44^

Reduced energy supply and metabolic alterations will program the fetus to the thrifty phenotype that favors survival, but may have an impact on development. The adaptation of the fetus to the adverse conditions during intrauterine growth restriction may alter its metabolism and physiology in an irreversible way. These changes alter the production and the release of hormones that modulate growth during fetal life, and thereby establish functional capacity and metabolic competence.^6^

## Endocrine Regulatory Axis and Central Nervous System

An adverse intrauterine environment is associated with metabolic disorders, but may also have repercussions in the development of the central nervous system (CNS).^55^ Therefore, the concept of the fetal origin of metabolic diseases in adults has been extended to chronic nonmetabolic diseases, such as psychiatric disorders. Cognitive deficits and behavioral problems, including hyperactivity and anxiety, have been inversely related to fetal growth.^56^ ^57^

Energy homeostasis is controlled by the CNS, and the peripheral indicators of energy balance include gastrointestinal neural signals due to the mechanical mechanism of chemoreceptors in the stomach and of metabolic signals detected in the blood, including insulin, glucose and leptin. These signals are integrated and processed in central autonomic control centers, located mainly in the brainstem and in the hypothalamus, leading to modulation of food intake and energy expenditure.^58^

The endocrine physiology related to the maintenance of energy homeostasis is profoundly altered by the effects of adipokines.^59^ Leptin, which is a central hormone for energy homeostasis, also has repercussions on neurodevelopment. Many peripheral and extrahypothalamic effects have been described, expanding the actions of leptin beyond energy balance.

The functions of this hormone were expanded to those of reproduction, glucose homeostasis, and proinflammatory mediator in immune system responses.^60^ Therefore, the hormonal axis glucose-insulin-IGF-1 and leptin regulates more than the energy stocks. Insulin-like growth factor 1, cytokines, and adipokines such as leptin may affect the development of the brain. These hormones are fundamental regulators involved in neuronal plasticity and have an impact on memory and on learning processes.^61^ ^62^

## Hormonal Biomarkers and Association with *Catch-up* Growth

The catch-up growth phenomenon has been thought of as an adaptative survival mechanism and refers to accelerated growth to compensate the intrauterine growth impairment.^63^ Besides the benefits of protecting the brain, this accelerated growth is associated with insulin resistance. The velocity of postnatal growth may be regulated by the hormonal insulin-IGF-1 axis and leptin.^64^ ^65^ ^66^ In the recovery of growth, the energy supply is mobilized to promote rapid growth, and favors the increase of fatty tissue stores—*catch-up fat*.^50^ The endocrine profile involves glucose intolerance, leptin resistance, and higher IGF-1 levels compared with infants without catch-up.^66^ ^67^ ^68^

The mechanisms of catch-up investigated after growth restriction showed alterations of central leptin signal and energy homeostasis, with increased resistance to leptin and to IGFs in children born small for gestational age.^69^ ^70^ During childhood, markers of cardiovascular risk, such as adiposity and leptin, were correlated.^71^ In adults who were growth-restricted newborns or preterm, markers of metabolic syndrome were detected, and insulin was associated with elevated blood pressure and triglycerides, and subsequent catch-up growth.^72^ ^73^

Fetal growth restriction was associated with the IGF system, with arterial hypertension, and with the risk of mortality in adulthood.^4^ ^74^ The alterations in the growth of the kidneys modify fetal programming, and may progress to cardiac dysfunctions.^75^ The association between IGF-1 and the severity score in cardiological clinical practice has been demonstrated.^76^ In childhood, biomarkers of cardiovascular diseases are associated with growth and with the accumulation of fat. Neonatal adiposity, when associated with pregnant women who have hyperglycemia and high levels of leptin in pregnancy, identifies children at risk of developing obesity and diabetes.^77^ Infants who were small for gestational age with accelerated growth have high risks for diabetes or cardiovascular diseases, regardless of their adiposity.^65^ ^68^ These findings have implications for the interpretation of pediatric adiposity indexes and of the trajectory of postnatal growth.

## Programming, hormonal biomarkers and clinical approach

Advances in knowledge about the pathophysiology of these diseases, which confirm that hormonal biomarkers affect growth in childhood, suggest new approaches in clinical follow-up. There is a recommendation for the evaluation of blood pressure, lipid, glucose, insulin, and IGF-1 levels in neonates with growth restriction. In the therapeutic approach, it is important to monitor these parameters. Most of these newborns showed completed catch-up growth up to the age of two years old.^78^ ^79^ Newborns with fetal growth restriction exposed to maternal gestational disease such as hypertension may develop the same maternal pathologies by previous exposure to the risk factors of metabolic disorders.^63^ ^80^ Leptin and IGF-1 may be predictive for newborns that will make the catch-up growth, and thus could be characterized as biomarkers of growth and as a prognostic tool in the neonatal clinic. These hormonal axis that are reset in the intrauterine period due to maternal pathologies, and which are also detected in human milk, remain to be elucidated in childhood.^81^ ^82^

**Fig. 1:**
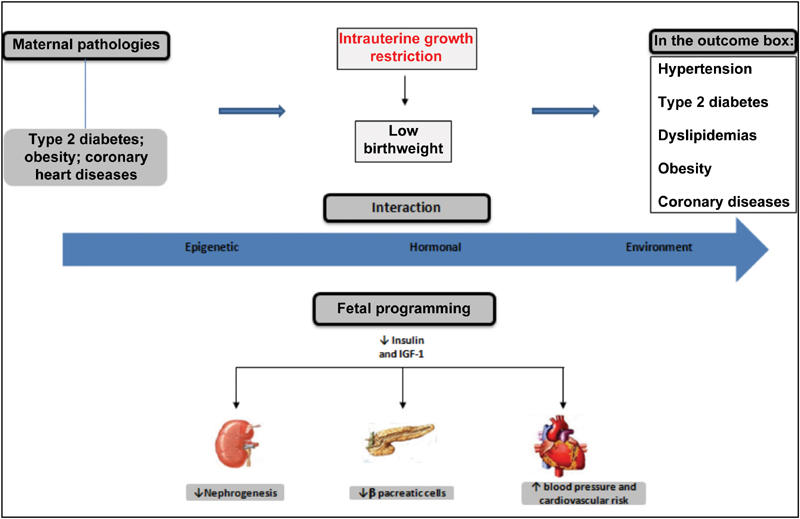
Fetal programming of chronic adult diseases.

The close interaction between fetal and maternal environment modulates gene expression, and these epigenetic modifications, including DNA methylation, mediate genomic imprinting. This phenomenon emerged as the molecular explanation of fetal metabolic programming, which persists in subsequent generations. The markers of both maternal undernutrition, and of maternal diabetic or obesity, such as impaired glucose tolerance, have been associated with elevated risk in the offspring.^83^ ^84^ Maternal pathologies such as hypertension and type 2 DM can affect the fetal endocrine axis and alter the trajectory of fetal growth. Infants of diabetic or of hypertensive mothers are prone to various neonatal adverse outcomes and are thought to be at high risk for metabolic diseases in adulthood.^83^ ^84^ ^85^ A summary diagram that guides the readers as to how these complex metabolic networks are interconnected and may be disturbed is shown in Fig. 1. The fetal hormonal alterations due to maternal pathologies can perpetuate a vicious cycle in the programming of fetal development. These metabolic disorders affect the development of the infant and can be transmitted across generations.^10^ ^86^

## Conclusion

Metabolic and morphological alterations early in the development promote fetal adaptation to adverse conditions. The plasticity and functional capacity of organs and systems occur in the critical period of intrauterine rapid growth. Fetal programming may be altered in response to environmental changes due to the action of hormones that regulate growth and can be transmitted across generations. The identification of biomarkers of fetal or postnatal programming could contribute to clinical monitoring, and provides new therapeutics targets to promote the healthy growth and development in childhood.
